# Characterization of a fluorescence imaging probe that exploits metabolic dependency of ovarian clear cell carcinoma

**DOI:** 10.1038/s41598-023-47637-0

**Published:** 2023-11-20

**Authors:** Saki Tsuchimochi, Osamu Wada-Hiraike, Yasuteru Urano, Asako Kukita, Kohei Yamaguchi, Harunori Honjo, Ayumi Taguchi, Michihiro Tanikawa, Kenbun Sone, Mayuyo Mori-Uchino, Tetsushi Tsuruga, Katsutoshi Oda, Yutaka Osuga

**Affiliations:** 1 Department of Obstetrics and Gynecology, Graduate School of Medicine, The University of Tokyo, Bunkyo, Tokyo, 113-8655 Japan; 2 Graduate School of Pharmaceutical Sciences, The University of Tokyo, Bunkyo, Tokyo, 113-0033 Japan; 3 CREST, Japan Agency for Medical Research and Development, Chiyoda, Tokyo, 100-0004 Japan; 4 Department of Integrated Genomics, Graduate School of Medicine, The University of Tokyo, Bunkyo, Tokyo, 113-8655 Japan

**Keywords:** Cancer, Cancer imaging, Tumour biomarkers

## Abstract

The purpose of this study is to clarify the metabolic dependence of ovarian clear cell carcinoma (CCC) by comparing normal tissues and to examine the applicability of fluorescence imaging probe to exploit these metabolic differences. Enhanced glutathione synthesis was supported by the increased uptake of related metabolites and elevated expression levels of genes. Accumulation of intracellular iron and lipid peroxide, induction of cell death by inhibition of the glutathione synthesis pathway indicated that ferroptosis was induced. The activation of γ-glutamyl hydroxymethyl rhodamine green (gGlu-HMRG), a fluorescent imaging probe that recognizes γ-glutamyl transferase, which is essential for the synthesis of glutathione, was investigated in fresh-frozen surgical specimens. gGlu-HMRG detected extremely strong fluorescent signals in the tumor lesions of CCC patients, compared to normal ovaries or endometrium. These results revealed that CCC occurs in the stressful and unique environment of free radical-rich endometrioma, and that glutathione metabolism is enhanced as an adaptation to oxidative stress. Furthermore, a modality that exploits these metabolic differences would be useful for distinguishing between CCC and normal tissues.

## Introduction

Epithelial ovarian cancer is classified into five histologic subtypes: high-grade serous (HGSOC), low-grade serous, mucinous carcinoma, endometrioid carcinoma (EC), and clear cell carcinoma (CCC), with HGSOC being the most common subtype. The subtypes differ greatly in their molecular characteristics^1^. Most HGSOC cases respond to conventional platinum-based chemotherapy, whereas the response rate of CCC is substantially lower than that of HGSOC^2^. The prognosis for advanced or recurrent cancer is extremely poor. Stages and residual diseases have been reported as prognostic factors for CCC^3,4^. Because of chemotherapy resistance, complete resection is an important treatment strategy. However, with current surgery relying on visual inspection and palpation by the operator, it is difficult to accurately detect cancerous tissues and distinguish them from normal tissues.

Because CCC occurs in an extremely stressful and unique environment of free radical-rich endometrioma, we hypothesized that there are metabolic differences between ovarian cancer and normal tissues, mainly in the oxidative stress tolerance mechanism. Endometriomas, the natal site of CCC, are formed by repeated local bleeding of an ectopic endometrium implanted within the ovary. Within the cyst, the accumulation of hemoglobin-derived iron generates reactive oxygen species (ROS) and antioxidant pathways (notably the glutathione pathway), which are induced to avoid cell damage by high levels of ROS^5^. In addition, the accumulation of lipid peroxide, which is produced by the oxidation of polyunsaturated fatty acid in membrane phospholipids, causes iron-dependent programmed cell death, known as ferroptosis^6,7^. Endometriomas have a mechanism for ferroptosis resistance that includes redox regulation by glutathione (GSH) metabolism, which allows the implantation and proliferation of endometriotic cells^8^. However, the accumulation of DNA damage that exceeds cellular repair capacity possibly causes CCC^5,9^. Previous studies have reported that CCC acquires oxidative stress tolerance at the genomic and transcriptomic levels as it develops and proliferates in a hypoxic environment with high ROS levels^9,10^. Although recent proteome analyses have shown enriched lipid metabolism and high expression of metabolic enzymes in the oxidative response pathways^4^, few reports on the detailed analyses of CCC’s metabolic state exist; therefore, further studies are required.

Fluorescence-guided surgery has been reported to be more sensitive than visual inspection or palpation and enables quicker evaluation of larger surfaces than histopathological examination using frozen sections^11^. γ-glutamyl hydroxymethyl rhodamine green (gGlu-HMRG) is a fluorescent probe that detects γ-glutamyl transferase (GGT) expression^12^. GGT is upregulated in many cancers, and the potential use of gGlu-HMRG via topical administration has been reported in various human cancer tissues, including lung^13^, head and neck^14^, liver^15^, papillary thyroid^16^, and breast cancer^17^. GGT is the only enzyme that cleaves the γ-glutamyl bond in GSH. Therefore, GGT is essential for GSH synthesis and is highly expressed when GSH production is increased. Fluorescence imaging studies using gGlu-HMRG have been reported in ovarian cancer using HGSOC and EC cell lines^12^; however, no studies have reported using CCC cell lines or clinical samples from patients with ovarian cancer.

To the best of our knowledge, this is the first report on fluorescence imaging probe using the unique metabolic state of CCC.

## Results

### Increased glutathione metabolism in CCC

**Figure 1:**
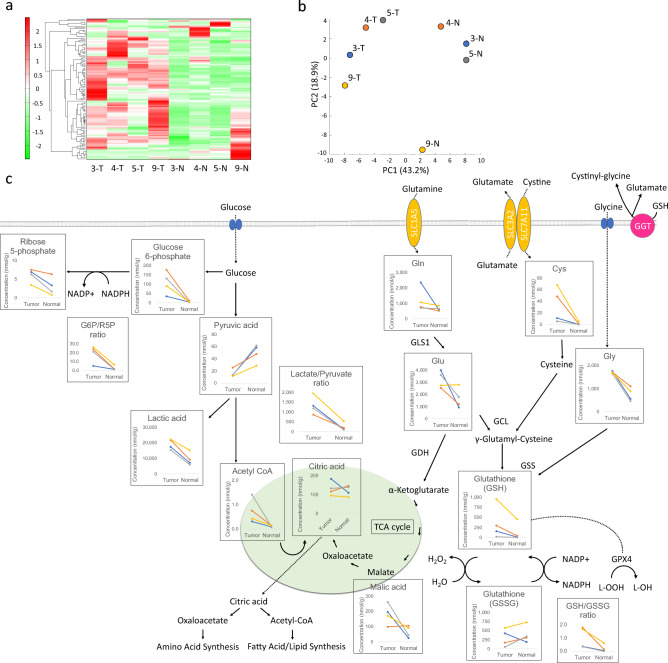
Comparison of CCC and normal ovarian tissues by metabolic analysis. Heatmap analysis showing metabolite profiles of each sample (**a**). Hierarchical clustering analysis (HCA) was performed using the metabolites detected in this study, and the results are shown in the heatmap display. The *x*-axis indicates the sample name, and the *y*-axis indicates the peak. HCA was performed on the peaks, and the distance between the peaks is shown using a tree diagram. Dark green indicates that the sample is smaller than the average, and dark red indicates that the sample is larger than the average. Principal component analysis (PCA) of the metabolome analysis (**b**); PC1 and PC2 indicate the first and second principal component scores, respectively, and the numbers in parentheses indicate the percentage contributions of each principal component. Schema of GSH metabolism and associated pathways (**c**). Absolute concentrations, lactate/pyruvate ratio, G6P/R5P ratio, and GSH/GSSG ratio for each metabolite in tumor and normal ovarian tissues are shown. Line colors in the graph are indicated in blue for case 3, orange for case 4, gray for case 5, and yellow for case 9.

Metabolomic analysis was performed using tumor and contralateral normal ovarian tissues from four patients (Table 1). A total of 116 compounds were analyzed. As shown in Fig. 1a, the results of the Hierarchical cluster analysis (HCA) showed differences in profiles between the tumor and normal ovarian tissue groups. High tumor and low normal ovarian tissue populations contained numerous amino acids, glycolytic metabolites, and pentose phosphate pathway metabolites. Pyruvic acid, unlike other glycolytic metabolites, was low in the tumor tissue group and high in the normal ovarian tissue group. Principal component analysis (PCA) showed that the tumor and normal ovarian tissue groups were well separated in the first principal component, and a difference between the two groups was observed. The scores of the first principal components are arranged in the order 9-T, 3-T, 4-T, 5-T, 9-N, 4-N, 3-N, and 5-N from smallest to largest (Fig. 1b). Metabolites with a correlation coefficient ≥ 0.9 were selected from the factor loading, which is defined by the correlation coefficient between the principal component score and each metabolite level. Metabolites that showed an increasing pattern had positive high loading values. Pyruvic acid was the only metabolite with a correlation coefficient > 0.9. Negative loading values indicate metabolites that show a decreasing pattern in normal ovaries compared to tumors. There were 48 metabolites with correlation coefficients > 0.9, including lactic acid and the amino acids glycine (Gly), glutamate (Glu), and cysteine (Cys), which are GSH components. Hypothesis testing using factor loadings from the PCA showed that Gln (*P* = 0.0002), Glu (*P* = 0.0193), Cys (*P* = 0.0439), lactic acid (*P* = 0.0005), and pyruvic acid (*P* = 0.0005) had significant correlations.

**Table 1:** Patient characteristics.

	Age	FIGO stage	T	N	M	Collection sites of the control
Case 1	67	IC3	1c3	0	0	Contralateral normal ovarian tissue
Case 2	52	IA	1a	0	0	Contralateral ovarian endometrioma, uterine endometrium
Case 3	48	IIB	2b	0	0	Contralateral normal ovarian tissue, uterine endometrium
Case 4	68	IIB	2b	0	0	Contralateral normal ovarian tissue
Case 5	29	IC3	1c3	0	0	Ipsilateral normal ovarian tissue, uterine endometrium
Case 6	68	IC3	1c3	0	0	Contralateral normal ovarian tissue, uterine endometrium
Case 7	46	IC3	1c3	0	0	Contralateral normal ovarian tissue, uterine endometrium
Case 8	42	IA	1a	0	0	Contralateral ovary with endometriosis
Case 9	51	IIIA2	3a	1	0	Contralateral normal ovarian tissue
Case 10	42	1C1	1C1	0	0	Contralateral ovary with endometriosis
Case 11	56	IC1	IC1	0	0	Contralateral normal ovarian tissue
Case 12	47	IVB	2b	0	1b	Contralateral normal ovarian tissue

*FIGO* International Federation of Gynecology and Obstetrics.

As shown in Fig. 1c, intracellular GSH is actively transported out of the cell, and extracellular GSH is degraded by GGT and incorporated into the cell as its constituent amino acids. Glutamine enters the cell via the amino acid transporter ASCT2/SLC1A5 and is converted to Glu by a deamination reaction catalysed by glutaminase (GLS). Cysteine is absorbed by the cell via SLC7A11, and GCL catalyses the ligation of Glu and Cys. γ-glutamyl-cysteine synthesis is catalysed by GSS (glutathione synthase) to produce GSH^18^. Glutathione peroxidase 4 (GPx4) reduces lipid hydroperoxide (L-OOH) to lipid hydroxide (L-OH) by using reduced GSH as a co-substrate, thereby inhibiting lipid peroxide accumulation^19^.

PCA showed no significant correlation for GSH (correlation coefficient of 0.584); however, because of the large differences between cases, tumor and normal ovarian tissue were compared in individual cases. This comparison showed that the absolute concentration of GSH was higher in tumor tissue than that in normal ovarian tissue in all four cases. The GSSG (Glutathione oxidized) /GSH ratio was higher in normal ovarian tissue than that in tumor tissue in all four cases. The reason for the discrepancy in higher oxidative stress in normal ovaries remains undetermined. However, because metabolome analysis can determine the number of intracellular metabolites pooled at a given moment, it may be difficult to determine GSH pathway activation from the metabolome analysis results owing to the rapid rate of GSH metabolism. The synthesis and degradation of GSH have a significant effect on its intracellular concentration^20^. We examined whether enzymes involved in the GSH synthesis pathway were upregulated in CCC compared with those in normal ovarian tissues using RT-qPCR.

**Figure 2:**
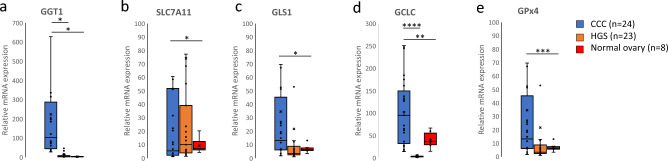
Comparison of expression levels of genes involved in GSH synthesis in CCC, HGSOC, and normal ovaries (**a**–**e**).

GGT1 (*P* = 0.009), SLC7A11 (*P* = 0.045), GLS1 (*P* = 0.02), GCLC (*P* < 0.001), and GPx4 (*P* < 0.001) were highly expressed in CCC compared with those in normal ovarian tissues (Fig. 2a–e). To confirm that enhanced GSH metabolism was specific to CCC, additional HGSOC samples were compared with CCC. In this comparison, the expression of GGT1 (*P* = 0.011) and GCLC (*P* < 0.001) were significantly higher in CCC (Fig. 2a and d). No significant differences in SLC7A11 (*P* = 0.655), GLS1 (*P* = 0.487), and GPx4 (*P* = 0.201) in comparison with HGSOC and CCC was observed (Fig. 2b,c,e).

### Accumulation of 4-HNE, a marker of oxidative stress

**Figure 3:**
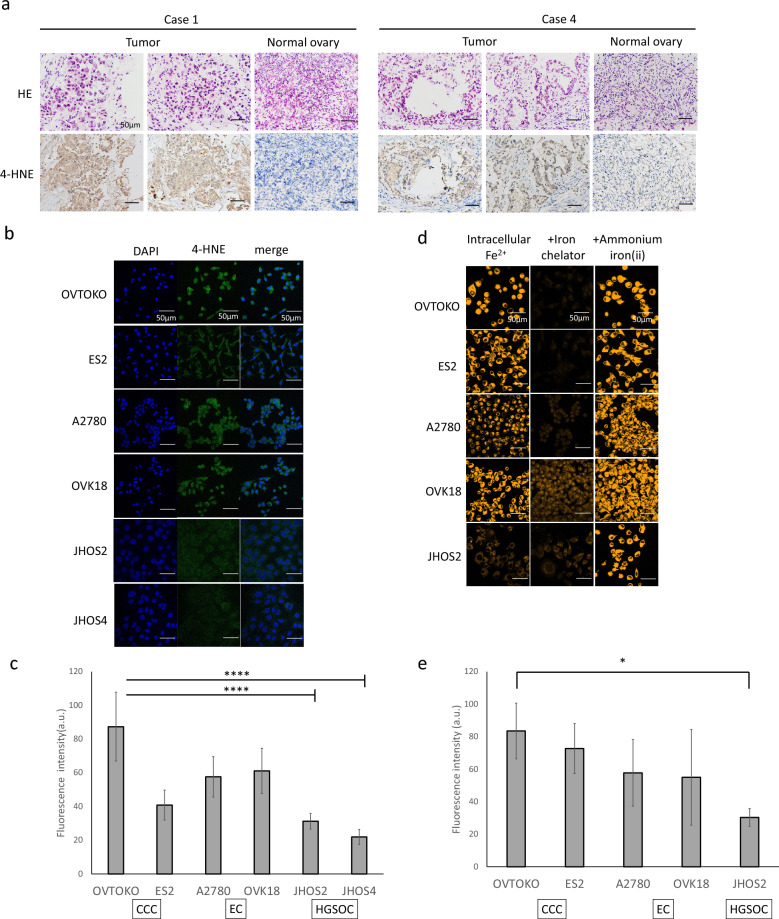
Lipid peroxide and intracellular iron accumulation in CCC. Representative images of CCC and normal ovary samples stained with H&E (top) and anti-4-HNE counterstained with hematoxylin (bottom) (**a**). Evaluation of 4-HNE accumulation in cell lines. Left: DAPI, center: 4-HNE, right: merge (**b**). Fluorescence intensity of 4-HNE (**c**); JHOS2 and JHOS4 had significantly lower fluorescence intensity than the 4 cell lines. Intracellular Fe^2+^ visualized by FerroOrange (left), addition of iron chelator (middle), and addition of ammonium iron (ii) (**d**). Intracellular Fe^2+^ fluorescence intensity in the basal line (**e**). Scale bar represents 50 µm.

4-hydroxynonenal (4-HNE), an aldehyde product of lipid peroxidation, is widely accepted as a stable marker of oxidative stress^21^. In the current study, immunohistological staining was performed to confirm the expression of 4-HNE in CCC. The results showed that 4-HNE expression was consistent with that observed in tumor tissues. Furthermore, the stromal and normal ovarian tissues were negative for 4-HNE (Fig. 3a). Next, fluorescent immunostaining of 4-HNE was performed on CCC, EC, and HGSOC cell lines (Fig. 3b), and fluorescence intensity was quantitatively evaluated (Fig. 3c). The two endometriosis-related cancers, CCC and EC, showed significantly higher fluorescence intensities than that of the two HGSOC cell lines (Supplementary Table 1).

### Detection of intracellular Fe^2+^ amount

Since the presence of intracellular iron is important for ferroptosis, intrinsic intracellular Fe^2+^ was confirmed using FerroOrange. The amount of intracellular Fe^2+^ was measured with and without the addition of the iron-chelating reagent Bpy (100 μmol/L 2,2′-bipyridine) and iron [100 μmol/L ammonium iron (II) sulphate] (Fig. 3d). In the CCC and EC cell lines, fluorescence was observed in the untreated state, but the addition of an iron chelating reagent decreased the fluorescence intensity, indicating the presence of endogenous Fe^2+^ in the cells. In contrast, fluorescence intensity was low in the unstimulated cells, and the addition of iron significantly increased the fluorescence intensity in the HGSOC cell lines. However, fluorescence was quenched by the addition of an iron-chelating reagent. Although only the OVTOKO and JHOS2 cell lines showed statistically significant differences (*P* = 0.04) (Supplementary Table 2), these results indicate that endogenous Fe^2+^ is significantly more abundant in endometriosis-related ovarian cancer, CCC, and EC than that in HGSOC (Fig. 3e).

### Ferroptosis is responsible for GSH/GPx4 inhibition-induced cell death

**Figure 4:**
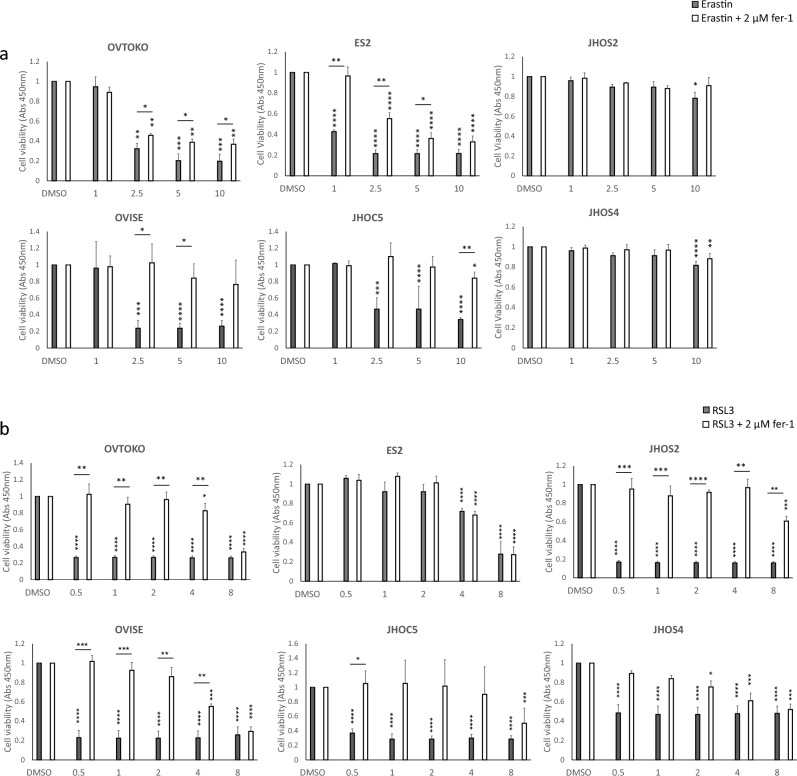
Inhibition of GSH/GPx4 pathway induces ferroptosis in CCC cell lines. The cells were treated with a stepwise dilution of erastin (**a**) or RSL3 (**b**) in the presence or absence of fer-1 for 72 h. Horizontal * indicates comparison of the presence or absence of fer-1, and vertical * indicates comparison at baseline and at each concentration.

The accumulation of lipid peroxide owing to GPx4 disruption and GSH depletion can cause ferroptosis^6^. Therefore, we investigated whether the inhibition of cystine uptake by the cystine/glutamic acid transporter inhibitor erastin induces ferroptosis. In CCC, elastin significantly decreased cell viability in a dose-dependent manner, whereas the addition of the ferroptosis inhibitor, ferrostatin-1 (fer-1), partially restored cell viability. In contrast, HGSOC did not show a significant decrease in viable cells up to 5 μM, but a decrease was observed at 10 μM (Fig. 4a). Next, we performed the same experiment using RSL3 ((1S,3R)-RSL3), an inhibitor of GPx4. RSL3-induced decrease in cell viability was observed in both CCC and HGSOC. Fer-1 treatment partially restored cell viability (Fig. 4b). Direct activation of ferroptosis by downregulating the GPx4 protein caused cell death in both CCC and HGSOC. However, only CCC was sensitive to the indirect induction of ferroptosis via the inhibition of GSH synthesis by erastin.

### Live cell imaging with gGlu-HMRG

**Figure 5:**
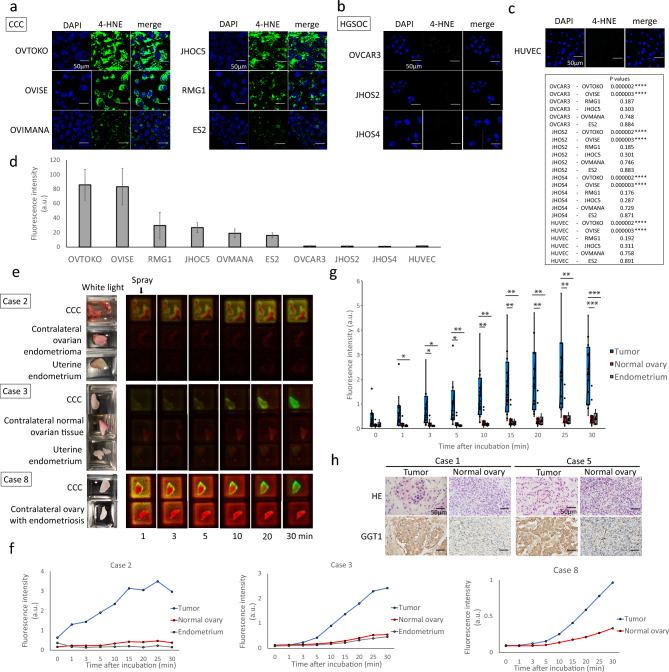
In vivo and ex vivo fluorescence imaging study of gGlu-HMRG. Fluorescence intensity of ovarian cancer cell lines 10 min after application of 2 µM gGlu-HMRG. Results are shown for CCC (**a**), HGSOC (**b**), and HUVEC (**c**). Fluorescence intensity of gGlu-HMRG (**d**). Results are expressed as mean ± SD (n = 3). Representative fluorescence images of fresh-frozen samples (**e**). The white light images before probe application and the color-coded fluorescence images based on wavelength spectrum after probe application are shown. Green indicates gGlu-HMRG, red indicates autofluorescence. A time course comparison of fluorescence intensity in CCC, normal ovary, and endometrium in Cases 2 and 3, CCC and normal ovary in Case 8 (**f**). Time course of fluorescence intensity in CCC, normal ovary and endometrium samples in 12 cases (**g**). The long bar indicates a comparison between tumor and endometrium, and the short bar indicates a comparison between tumor and normal ovary. Representative images of CCC and normal ovary samples stained with H&E (top), and anti-GGT1 counterstained with hematoxylin (bottom) (**h**). Scale bar represents 50 µm.

Urano et al. reported that 11 ovarian cancer cell lines exhibit activation and accumulation of the gGlu-HMRG probe^12^. However, several of these cell lines had low fluorescence intensity, resulting in failure to detect peritoneal dissemination in mice. Although CCC has a significantly higher expression of GGT1 compared to that of HGSOC, fluorescence could be detected more strongly in CCC than in HGSOC. Because there are no studies using CCC cell lines, we performed live cell imaging with gGlu-HMRG of CCC cell lines. HGSOC and HUVEC cell lines were used for comparison. Ten minutes after the addition of the fluorescent probe, all CCC cell lines showed green fluorescence (Fig. 5a), while HGSOC (Fig. 5b) and HUVEC (Fig. 5c) cell lines also showed an increase in fluorescence intensity, but it was markedly lower than that produced in CCC (Fig. 5d).

### Ex vivo fluorescence imaging study in clinical specimens

Subsequently, 50 µM gGlu-HMRG was sprayed on the clinical specimens of surgically excised CCC cases, and changes in fluorescence intensity over time were measured. Normal ovarian tissue was obtained from the contralateral normal ovary or contralateral ovarian endometrioma in patients who underwent bilateral adnexectomy, and from the ipsilateral normal ovary in patients who underwent unilateral adnexectomy. In five cases, the uterine endometrium was also sampled. The sites of sampling for each case are listed in Table 1. In all 12 cases, fluorescence in tumor lesions was detected within minutes and showed a remarkable increase in fluorescence intensity compared to that in normal tissues (Fig. 5e–g and Supplementary Fig. 1); the green fluorescence of gGlu-HMRG was visible to the naked eye. All cases were histologically confirmed to be CCC. Immunohistological staining was performed to confirm the expression of GGT protein in CCC lesions. The results showed that GGT expression was consistent with tumor lesions (Fig. 5h).

## Discussion

The purpose of this study was to clarify the metabolic dependence of ovarian CCC by comparing normal tissues and to examine the applicability of fluorescence imaging probe to exploit these metabolic differences. Enhanced GSH synthesis was supported by the increased uptake of amino acids and elevated expression levels of genes involved in GSH synthesis. Investigating the role of the GSH/GPx4 pathway on CCC, we found that inhibition of GSH synthesis and GPx4 induced ferroptosis, and removal of lipid peroxide restored cell viability, indicating that prevention of ferroptosis is strongly dependent on the GSH pathway. gGlu-HMRG detected extremely strong fluorescent signals in the tumor lesions of all 12 CCC patients, compared to signals obtained from normal ovaries or endometrium. These results support our hypothesis that CCC occurs in the extremely stressful and unique environment of free radical-rich endometrioma, and that GSH metabolism is enhanced as an adaptation to oxidative stress. Therefore, a modality that exploits these metabolic differences may be useful for distinguishing between CCC and normal ovaries.

CCC has been reported to occur in ovarian endometrioma that are associated with oxidative stress. Although the etiology of endometriosis is not fully understood, it is known that menstrual blood reflux into the pelvis and ovaries is involved in its development. Elevated iron levels generate ROS via inflammatory reactions, Fenton reactions, and autoxidation, leading to peroxidation of membrane phospholipids and induction of ferroptosis. However, ectopic endometriosis has a ferroptosis-resistant mechanism that prevents cell death by ferroptosis and allows endometriotic cells to implant and proliferate in the peritoneum and ovaries to form endometriosis lesions^8^. A study comparing differences in gene expression between eutopic and ectopic endometrium of endometriosis patients using big data including 164 expression arrays reported that ferroptosis resistance in endometriotic lesions is associated with iron, lipid, cholesterol, glucose metabolism, and redox regulation, including GSH metabolism^5^. Although this study reported that genetic mutations involved in the above metabolism accumulate during the process from eutopic to ectopic, there has been no detailed examination of how metabolism is altered by the genetic mutations that occur during the process of ectopic endometriosis to cancer^5^.

It is crucial to compare CCC with endometriosis to elucidate the metabolism of CCC. Comparison with ovarian endometriomas is appropriate for metabolomic analysis of CCC, but it is difficult to collect the necessary amount of endometriotic tissue for analysis, so contralateral normal ovarian tissue without endometriosis was used in this study. However, for the ex vivo fluorescence imaging study in clinical specimens, ovarian endometriomas and uterine endometrium were used as normal tissue controls, allowing comparison of gGlu-HMRG activity. Although CCC develops and proliferates in a hypoxic environment with high levels of ROS, we did not conduct any experiment under hypoxia. The potential effects of hypoxia on GGT1 expression and gGlu-HMRG activation need to be explored. While iron import, export, storage, and turnover are known to affect susceptibility to ferroptosis, our study only evaluated intracellular iron accumulation and did not assess dynamic iron metabolism, including transferrin, transferrin receptors, and ferritinophagy. In addition, this study only showed the accumulation of lipid peroxide by accumulation of 4-HNE, a metabolite of lipid peroxide, and did not examine lipid metabolism in detail. To further understand ferroptosis in CCC, measurements of ROS levels and 4-HNE in response to iron chelation and iron treatment are needed.

Many studies have been conducted to elucidate the relationship between mutations in oncogenes and tumor suppressors and the resulting changes in metabolic pathways, with potential therapeutic applications^22^. In general, however, it is difficult to target metabolic abnormalities observed in tumor cells for therapy. The reason for this is the existence of metabolic pathways that bypass the inhibition point and overlap with normal cell metabolism. Teragrenastat (CB-839) is an inhibitor of glutaminase (GLS1), an enzyme that catalyzes the deamidation of glutamine to glutamic acid. Teragrenastat has shown efficacy in several preclinical glutamine-dependent cancer models, including triple-negative breast cancer^23^, non-small cell lung cancer^24^, acute myeloid leukemia^25^ and renal cell carcinoma^26,27^. In ovarian cancer, overexpression of GLS1 has been reported in patients with recurrent ovarian cancer previously treated with platinum^28^. A phase I trial is currently ongoing to evaluate the efficacy of CB-839 in combination with niraparib in patients with platinum-refractory wild-type BRCA ovarian cancer. Inactivation of GLS impairs redox balance, inhibits nucleotide synthesis, causes replication stress, and induces DNA repair in cancer cells. Notably, CB-839 is expected to be particularly effective in combination with PARP inhibitors^22^. Ovarian cancers refractory to standard platinum-based chemotherapy may benefit from treatment with this glutaminase inhibitor, and the results of clinical trials are pending. However, few attempts have been made to use metabolic dependencies in cancer cells for diagnosis.

Fluorescence-guided surgery is useful for the resection of tumors with sufficient tumor-free margins, detection of clinically occult lesions, and debulking procedures to remove as much of the visualized tumor as possible^11^. Fluorescence-guided neurosurgery using 5-aminolevulinic acid^29–31^ and hepatic resection using indocyanine green^32,33^ have been implemented. A phase III trial of intraoperative imaging using near-infrared light (NIR) with injectable pafolacianine (OTL38) for folate receptor-positive ovarian cancer was recently conducted. Efficacy was defined as the percentage of patients in whom fluorescent imaging yielded at least one additional tumor-positive lesion compared with normal conditions and reported the usefulness of pafolacianine as a real-time adjuvant diagnosis^34^. As most patients in the clinical study had serous carcinomas and only a few percent had CCC^34,35^, whether usefulness of pafolacianine for CCC is not known. Although the metabolomic analysis in this study was performed in a small number of cases (4 tumors and 4 normal ovarian tissues), no significant differences were found in folate concentrations. OTL38 requires intravenous injection at least one hour prior to imaging, whereas gGlu-HMRG is superior because it is sprayed directly onto the lesion and fluoresces within minutes. In addition, the fluorescent probe can be removed by washing after observation, it is considered to have little effect on the human body. Also, the advantages of gGlu-HMRG against NIR fluorophore is that NIR signal is not visible to the naked eye, but a gGlu-HMRG signal does not require any special equipment if we see the signal without quantification, and the signal can be observed with the naked eye through a blue filter. Although only fresh-frozen tissue samples were used in this study, we would like to apply this technique to fluorescence-guided surgery in the future. Finally, as the goal of fluorescence-guided surgery is to improve patient survival, future studies are needed to determine whether resection of additional lesions by fluorescence imaging improves long-term prognosis.

This is the first study to report fluorescence imaging probe using the unique metabolic state of cancer cells. The metabolism of the GSH pathway in CCC has been shown to be related to the prevention of ferroptosis. This result supports the usefulness of gGlu-HMRG in detecting CCC lesions. The gGlu-HMRG probe may be a novel modality for optimal debulking surgery because of its rapid, strong, and highly selective activation upon contact with GGT.

## Methods

### Activatable fluorescence probe

gGlu-HMRG is a fluorescent probe used to detect GGT activity. It reacts irreversibly with GGT, changing into a green, fluorescent material, which is highly expressed on the surface of cancer cells. This green material is rapidly absorbed by intracellular lysosomes, allowing the detection of cancer tissues that express GGT^12^. A 10 mM dimethyl sulfoxide (DMSO) stock solution of gGlu-HMRG was provided by Urano and diluted with Hanks' Balanced Salt Solution (HBSS; AAT Bioquest, Sunnyvale, CA, USA; Cat #20011) to the final concentration described in the experimental section.

### Cell lines and culture conditions

OVISE (RRID: CVCL_3116), OVMANA (RRID: CVCL_3111), OVTOKO (JCRB Cat# JCRB1048, RRID: CVCL_3117), and RMG1 (RRID: CVCL_1662) were purchased from Japanese Cancer Research Resources Bank (JCRB). OVCAR3 (RRID: CVCL_0465), JHOC5 (RRID: CVCL_4640), JHOS2 (RRID: CVCL_4647), and JHOS4 (RRID: CVCL_4649) were purchased from RIKEN Cell Bank. ES-2 (RRID: CVCL_3509) was purchased from the American Type Culture Collection (ATCC). OVISE, OVMANA, OVTOKO, and OVCAR3 cells were cultured in RPMI-1640 medium (Fujifilm Wako Pure Chemical Corporation, Osaka, Japan; Cat# 189-02145) containing 10% fetal bovine serum (FBS; Gibco, MA, USA; Cat# 10270106) and 1% penicillin/streptomycin (Fujifilm Wako Pure Chemical Corporation; Cat# 161-23181). RMG1 cells were cultured in Ham’s F-12 medium (Fujifilm Wako Pure Chemical Corporation; Cat# 087-08335) supplemented with 10% FBS and 1% penicillin/streptomycin. ES-2 cells were cultured in McCoy's 5A (modified) medium (Gibco/Invitrogen, Carlsbad, CA, USA; Cat# 16600082), containing 10% FBS and 1% penicillin/streptomycin. JHOC5, JHOS-2, and JHOS-4 cells were cultured in DMEM/HamF12 (Fujifilm Wako Pure Chemical Corporation; Cat# 048-29785) supplemented with 10% FBS, 0.1 mM non-essential amino acids (Cytiva, Issaquah, WA; Cat #SH30238.01), and 1% penicillin/streptomycin. Human umbilical vein endothelial cells (HUVECs) were purchased from ScienCell (San Diego, CA, USA; Lot# 28433) and cultured in endothelial cell growth medium (Takara Bio, Shiga, Japan; Cat# C-22010) with 1% penicillin/streptomycin. All the cell lines were cultured in a humidified atmosphere at 37 °C. Cells were routinely tested for mycoplasma using the MycoAlert Mycoplasma Detection Kit (Lonza, Walkersville, MD, USA; Cat# LT07-118).

### Clinical samples

Fresh-frozen tissue samples were obtained from patients who underwent surgery for ovarian cancer at the Department of Gynecologic Surgery, Graduate School of Medicine, University of Tokyo, from 2021 to 2022. Immediately after excision, tissue samples were macroscopically cut into small pieces, frozen in liquid nitrogen, and preserved at − 80 °C until its analysis. All procedures were performed in accordance with the protocols approved by the Research Review Board at our institution (no. 2022008NI) and signed informed consent for the use of the tissues was obtained from each patient.

### Metabolite extraction

Approximately 40 mg of frozen tissue was put into a homogenization tube along with zirconia beads (5 mm diameter and 3 mm diameter). Next, 750 µL of 50% acetonitrile/Milli-Q water containing internal standards [H3304-1002, Human Metabolome Technologies, Inc. (HMT), Yamagata, Japan] was added to the tube. The tissue was then shaken 20 times at 3500 rpm and 4 °C for 60 s using a bead shaker (Shake Master NEO, Bio Medical Science, Tokyo, Japan). and centrifuged at 2300×*g* at 4 °C for 5 min to obtain the homogenate. Subsequently, the upper aqueous layer was centrifugally filtered through a Millipore 5-kDa cutoff filter (UltrafreeMC-PLHCC, HMT) at 9100×*g* and 4 °C for 120 min to remove macromolecules. The filtrate was dried by evaporationunder vacuum and reconstituted in 50 µL of Milli-Q water for metabolomic analysis at HMT.

### Metabolome analysis (C-SCOPE)

Metabolome analysis was conducted according to HMT’s *C-SCOPE* package, using capillary electrophoresis time-of-flight mass spectrometry (CE-TOFMS) for cation analysis and CE-tandem mass spectrometry (CE-MS/MS) for anion analysis, based on previously described methods^36,37^. Peaks were extracted using MasterHands, automatic integration software (Keio University, Yamagata, Japan)^38^ and Agilent Masshunter Quantitative Analysis software (Agilent Technologies, Santa Clara, CA, USA, RRID:SCR_015040) to obtain peak information including *m/z*, peak area, and migration time (MT). Signal peaks were annotated according to the HMT metabolite database based on their *m*/*z* values and MTs. The peak area of each metabolite was normalized to the internal standards, and metabolite concentrations were evaluated using standard curves with three-point calibrations using each standard compound. HCA and PCA^39^ were performed HMT’s proprietary MATLAB (RRID:SCR_001622) and R Project for Statistical Computing (RRID:SCR_001905) respectively.

### RNA extraction and RT-qPCR

Fresh-frozen tissue samples were homogenized using a MagNA Lyser Instrument (Roche Diagnostics, Tokyo, Japan; Cat# 03358968001) and MagNA Lyser Green Beads (Roche Diagnostics; Cat# 03358941001). Total RNA was extracted using the RNeasy Mini Kit (Qiagen, Hilden, Germany; Cat# 74104), and cDNA was synthesized from 2 μg of Total RNA using qPCR RT Master Mix with gDNA Remover (Toyobo, Osaka, Japan; Cat# FSQ-301), according to the manufacturer's protocol. RT-qPCR was performed using KOD SYBR qPCR Mix (Takara Bio; Cat #QKD-201 X5) with the following primers (Supplemental Table 3), and the amplified cDNA was analyzed using the Applied Biosystems QuantStudio 1 RealTime PCR System (RRID:SCR_023003).

### Immunohistochemistry

Formalin-fixed, paraffin-embedded (FFPE) tissue blocks were sectioned at 4 μm thickness. These sections were deparaffinized in xylene, rehydrated through graded alcohols, and then immersed in Target Retrieval Solution, Citrate pH 6 (Agilent Dako, Santa Clara, CA, USA; Cat# S2369) for heat-induced antigen retrieval. Endogenous peroxidase activity was blocked with a peroxidase-blocking solution (Agilent Dako; Cat# S202386-2). Bovine serum albumin (BSA; 1%) was used to block nonspecific reactions. The sections were incubated with a 1:1000 dilution of Mouse Anti-4-Hydroxynenal Monoclonal antibody (R and D Systems, Minneapolis, MN, USA; Cat# MAB3249, RRID: AB_664165) and 1:100 dilution of anti-GGT1 Antibody (LSBio (LifeSpan), Seattle, WA, USA; Cat# LS-B5416-50, RRID: AB_10944411) overnight at 4 °C. The antigen–antibody reactions were detected with EnVision + Dual Link System-HRP (Agilent Dako; Cat# K4063) and visualized using the UltraView Universal DAB Detection Kit (Ventana Medical Systems, Tucson, AZ, USA; Cat# 760-500). The sections were counterstained with 10% Mayer’s hematoxylin (Fujifilm Wako Pure Chemical Corporation; Cat# 131-09665), dehydrated, and mounted.

### Immunofluorescence-immunocytochemistry

Cells plated on coverslips were pre-extracted with Cytoskeletal Buffer and then cells were fixed in 4% paraformaldehyde for 15 min at room temperature. After fixation, cells were washed twice with 0.05% Tween 20 in PBS and permeabilized with 0.5% Triton X-100 in PBS for 10 min. After blocking with 3% BSA for 60 min, the cells were incubated overnight at 4 °C in a wet chamber with 1 μg/mL Mouse Anti-4-Hydroxynenal Monoclonal antibody (R and D Systems Cat# MAB3249, RRID: AB_664165) diluted with 3% BSA. The cells were again washed twice with 0.05% Tween 20 in PBS and permeabilized with 0.5% Triton X-100 in PBS for 10 min. After washing with 0.05% Tween 20 in PBS, the nuclei were stained with 4′,6-diamidino-2-phenylindole (DAPI) for 5 min, washed with distilled water, mounted with ProLong Gold (Life Technologies, Carlsbad, CA, USA), and visualized using a confocal laser-scanning microscope (Carl Zeiss LSM 700, Oberkochen, Germany). Image analysis was performed using Fiji (RRID:SCR_002285)^36^.

### Detection of intracellular Fe^2+^ amount

A total of 5 × 10^3^ cells were seeded in 8-well chamber slides (Ibidi, Munich, Germany; Cat# ib80826) and incubated for 24 h at 37 °C and 5% CO_2_, after which they were washed thrice with serum-free medium. Thereafter, the cells were incubated for 20 min in the presence or absence of 100 μM ammonium iron (II) sulphate. After incubation, cells were washed thrice with HBSS and stained with 1 μM Ferro orange (Dojindo Molecular Technologies, Kumamoto, Japan; Cat# 342-09533) with or without 100 μM 2,2′-bipyridyl (Bpy) for 30 min at 37 °C and 5% CO_2_. The cell-staining solution was replaced with HBSS prior to observation. Fluorescence images were captured using a confocal laser-scanning microscope (Zeiss LSM 700). A TRITC filter set (ex, 561 nm; em 570–620) was used. Image analysis was performed using Fiji (RRID: SCR_002285)^36^.

### Inhibition of GSH synthesis induces ferroptosis

Cells from each cell line were treated with the step dilution of erastin or solvent (DMSO) either in the presence or absence of 2 μM fer-1 for 72 h. Cell viability was calculated by using Cell Counting Kit-8 (Dojindo Molecular Technologies; Cat# 341-07761) according to the manufacturer’s instructions. The drugs used in these experiments were obtained as follows: erastin (HY-15763, MedChemExpress), (1S,3R)-RSL3 (19288, Cayman Chemical), fer-1 (17729, Cayman Chemical).

### Live cell imaging with gGlu-HMRG

Cells from each cell line were seeded on 8-well chamber slides (Ibidi; Cat# ib80826) at a density of 5 × 10^3^ cells per well and incubated for 48 h at 37 °C. gGlu-HMRG (2 μM) in HBSS (AAT Bioquest; Cat# 20011) was added to the cells and incubated at 37 °C and 5% CO_2_ for 10 min. Next, nuclei were stained with Hoechst 33342 (Dojindo Molecular Technologies; Cat# 346-07951). Thereafter, the cells were washed twice with HBSS, and fluorescence images were captured using a confocal laser-scanning microscope (Zeiss LSM 700). The excitation and emission wavelengths were 488 nm and 500–600 nm for gGlu-HMRG and 345 and 455 nm for Hoechst 33342, respectively. Image analysis was performed using Fiji (RRID: SCR_002285)^36^.

### Ex vivo fluorescence imaging study in clinical specimens

Fresh-frozen tissue samples were subjected to fluorescence imaging analysis after adjusting to 25 °C. Next, gGlu-HMRG was diluted with HBSS (AAT Bioquest; Cat# 20011) to 50 μM. Images were captured using a Maestro In Vivo Imaging System (CRi Inc., Toronto, ON, Canada) before and at 1, 3, 5, 10, 15, 20, 25, and 30 min after applying the gGlu-HMRG working solution. The excitation and emission wavelengths were 445–490 nm and 515 nm, respectively. Fluorescence images were extracted at 540 nm. Region of interests (ROIs) were set for both ovarian tumors and normal ovaries, and the maximum fluorescence intensity of each ROI was calculated using Maestro software. The increase in fluorescence intensity was calculated by subtracting the initial fluorescence intensity from that measured after each incubation period with gGlu-HMRG.

### Histological analysis

After fluorescence imaging, the specimens were immediately fixed in 4% paraformaldehyde for 24 h. Paraffin-embedded sections were stained with Hematoxylin and Eosin (H&E) for histopathological evaluation. An experienced pathologist examined each sample in a blinded manner to ensure that the tumor sections contained adenocarcinoma and normal ovarian or endometrial sections did not.

### Statistical analyses

All statistical analyses were performed using EZR (Saitama Medical Center, Jichi Medical University, Saitama, Japan), a graphical user interface for R software (R Foundation for Statistical Computing, Vienna, Austria; RRID: SCR_001905). EZR is a modified version of the R commander with statistical functions frequently used in biostatistics^37^. The experiments were performed in triplicate. All data were expressed as mean ± standard deviation. Normally distributed data were analyzed using two-tailed Student’s *t*-test and one-way ANOVA. Multiple tests after ANOVA were performed using Tukey’s multiple comparison test. Data that were not normally distributed and did not have homogeneity of variance were analyzed using Welch’s *t*-test and the Kruskal–Wallis test. Multiple testing after analysis of variance was performed using Steel–Dwass multiple testing. Statistical significance was set at *P < 0.05; **P < 0.01; ***P < 0.001; ****P < 0.0001.

### Ethics statement

Approval of the research protocol by an Institutional Reviewer Board: Ethical approval for this study was obtained from the Institutional Review Board of the University of Tokyo (no. 2022008NI).

### Informed consent

Signed informed consent for the use of the tissue was obtained from each patient.

### Supplementary Information

Supplementary Information.

## Data Availability

The datasets used or analyzed during the current study are available from the corresponding author on reasonable request.

## References

[1] Iida Y, Okamoto A, Hollis RL, Gourley C, Herrington CS (2021). Clear cell carcinoma of the ovary: A clinical and molecular perspective. Int. J. Gynecol. Cancer.

[2] Gadducci A (2021). Clear cell carcinoma of the ovary: Epidemiology, pathological and biological features, treatment options and clinical outcomes. Gynecol. Oncol..

[3] Sugiyama T (2000). Clinical characteristics of clear cell carcinoma of the ovary: A distinct histologic type with poor prognosis and resistance to platinum-based chemotherapy. Cancer.

[4] Ji JX (2022). The proteome of clear cell ovarian carcinoma. J. Pathol..

[5] Li B, Duan H, Wang S, Li Y (2021). Ferroptosis resistance mechanisms in endometriosis for diagnostic model establishment. Reprod. Biomed. Online.

[6] Stockwell BR (2017). Ferroptosis: A regulated cell death nexus linking metabolism, redox biology, and disease. Cell.

[7] Dixon SJ (2012). Ferroptosis: An iron-dependent form of nonapoptotic cell death. Cell.

[8] Ng SW, Norwitz SG, Taylor HS, Norwitz ER (2020). Endometriosis: The role of iron overload and ferroptosis. Reprod. Sci..

[9] Amano T, Murakami A, Murakami T, Chano T (2021). Antioxidants and therapeutic targets in ovarian clear cell carcinoma. Antioxidants.

[10] Ji JX, Wang YK, Cochrane DR, Huntsman DG (2018). Clear cell carcinomas of the ovary and kidney: Clarity through genomics. J. Pathol..

[11] Lauwerends LJ (2021). Real-time fluorescence imaging in intraoperative decision making for cancer surgery. Lancet Oncol..

[12] Urano Y (2011). Rapid cancer detection by topically spraying a gamma-glutamyltranspeptidase-activated fluorescent probe. Sci. Transl. Med..

[13] Hino H (2016). Rapid cancer fluorescence imaging using A gamma-glutamyltranspeptidase-specific probe for primary lung cancer. Transl. Oncol..

[14] Mizushima T (2016). Fluorescent imaging of superficial head and neck squamous cell carcinoma using a gamma-glutamyltranspeptidase-activated targeting agent: A pilot study. BMC Cancer.

[15] Miyata Y (2017). Intraoperative imaging of hepatic cancers using gamma-glutamyltranspeptidase-specific fluorophore enabling real-time identification and estimation of recurrence. Sci. Rep..

[16] Hino R (2018). Rapid detection of papillary thyroid carcinoma by fluorescence imaging using a gamma-glutamyltranspeptidase-specific probe: A pilot study. Thyroid Res..

[17] Ueo H (2015). Rapid intraoperative visualization of breast lesions with gamma-glutamyl hydroxymethyl rhodamine green. Sci. Rep..

[18] Lushchak VI (2012). Glutathione homeostasis and functions: Potential targets for medical interventions. J. Amino Acids.

[19] Zou Y (2019). A GPX4-dependent cancer cell state underlies the clear-cell morphology and confers sensitivity to ferroptosis. Nat. Commun..

[20] Baudouin-Cornu P, Lagniel G, Kumar C, Huang ME, Labarre J (2012). Glutathione degradation is a key determinant of glutathione homeostasis. J. Biol. Chem..

[21] Zhong H, Yin H (2015). Role of lipid peroxidation derived 4-hydroxynonenal (4-HNE) in cancer: Focusing on mitochondria. Redox Biol..

[22] Lemberg KM, Gori SS, Tsukamoto T, Rais R, Slusher BS (2022). Clinical development of metabolic inhibitors for oncology. J. Clin. Investig..

[23] Gross MI (2014). Antitumor activity of the glutaminase inhibitor CB-839 in triple-negative breast cancer. Mol. Cancer Ther..

[24] Caiola E (2020). Glutaminase inhibition on NSCLC depends on extracellular alanine exploitation. Cells.

[25] Matre P (2016). Inhibiting glutaminase in acute myeloid leukemia: Metabolic dependency of selected AML subtypes. Oncotarget.

[26] Raczka AM, Reynolds PA (2019). Glutaminase inhibition in renal cell carcinoma therapy. Cancer Drug Resist..

[27] Hoerner CR, Chen VJ, Fan AC (2019). The ‘Achilles heel’ of metabolism in renal cell carcinoma: Glutaminase inhibition as a rational treatment strategy. Kidney Cancer.

[28] Shen YA (2020). Inhibition of the MYC-regulated glutaminase metabolic axis is an effective synthetic lethal approach for treating chemoresistant ovarian cancers. Cancer Res..

[29] Cornelius JF (2014). Impact of 5-aminolevulinic acid fluorescence-guided surgery on the extent of resection of meningiomas–with special regard to high-grade tumors. Photodiagnosis Photodyn. Ther..

[30] Hollon T, Stummer W, Orringer D, Suero Molina E (2019). Surgical adjuncts to increase the extent of resection: Intraoperative MRI, fluorescence, and Raman histology. Neurosurg. Clin. N. Am..

[31] Foster N, Eljamel S (2016). ALA-induced fluorescence image guided surgery of meningiomas: A meta-analyses. Photodiagnosis Photodyn. Ther..

[32] Ishizawa T (2009). Real-time identification of liver cancers by using indocyanine green fluorescent imaging. Cancer.

[33] Purich K (2020). Intraoperative fluorescence imaging with indocyanine green in hepatic resection for malignancy: A systematic review and meta-analysis of diagnostic test accuracy studies. Surg. Endosc..

[34] Tanyi JL (2023). A Phase III study of Pafolacianine injection (OTL38) for intraoperative imaging of folate receptor-positive ovarian cancer (Study 006). J. Clin. Oncol..

[35] Randall LM, Wenham RM, Low PS, Dowdy SC, Tanyi JL (2019). A phase II, multicenter, open-label trial of OTL38 injection for the intra-operative imaging of folate receptor-alpha positive ovarian cancer. Gynecol. Oncol..

[36] Schindelin J (2012). Fiji: An open-source platform for biological-image analysis. Nat. Methods.

[37] Kanda Y (2013). Investigation of the freely available easy-to-use software 'EZR' for medical statistics. Bone Marrow Transplant..

[38] Sugimoto M, Wong DT, Hirayama A, Soga T, Tomita M (2010). Capillary electrophoresis mass spectrometry-based saliva metabolomics identified oral, breast and pancreatic cancer-specific profiles. Metabolomics.

[39] Yamamoto H (2014). Statistical hypothesis testing of factor loading in principal component analysis and its application to metabolite set enrichment analysis. BMC Bioinform..

