# Supplementary material for: Characterization of a fluorescence imaging probe that exploits metabolic dependency of ovarian clear cell carcinoma
Source: Sci Rep. 2023 Nov 20;13:20292. doi: 10.1038/s41598-023-47637-0 (PMC10662153; doi:10.1038/s41598-023-47637-0)
Supplement: Supplementary file 1 — Supplementary Information. [file 41598_2023_47637_MOESM1_ESM.pdf]

Supplementary Table 1

Results of Steel-Dwass multiple testing for fluorescent immunostaining of 4-HNE.

	t	p
A2780-ES2	8.98	4.10E-14
A2780-JHOS2	8.60	5.35E-14
A2780-JHOS4	4.94	1.16E-05
A2780-OVK18	1.70	5.34E-01
A2780-OVTOKO	8.19	7.46E-14
ES2-JHOS2	6.03	2.53E-08
ES2-JHOS4	4.68	4.12E-05
ES2-OVK18	10.28	6.39E-14
ES2-OVTOKO	10.21	5.28E-14
JHOS2-JHOS4	3.89	1.42E-03
JHOS2-OVK18	8.87	4.34E-14
JHOS2-OVTOKO	7.97	1.05E-13
JHOS4-OVK18	4.97	9.77E-06
JHOS4-OVTOKO	4.77	2.77E-05
OVK18-OVTOKO	7.58	5.40E-13

Supplementary Table 2

Results of Tukey's multiple comparison test for detection of intracellular Fe^{2+}.

	diff.mean	lwr	upr	adjusted p-value
ES2-A2780	14.95	-36.68	66.57	0.87
JHOS2-A2780	-27.46	-79.08	24.16	0.45
OVK18-A2780	-2.79	-54.42	48.83	1.00
OVTOKO-A2780	25.75	-25.88	77.37	0.51
JHOS2-ES2	-42.40	-94.03	9.22	0.12
OVK18-ES2	-17.74	-69.36	33.88	0.79
OVTOKO-ES2	10.80	-40.82	62.42	0.95
OVK18-JHOS2	24.66	-26.96	76.29	0.54
OVTOKO-JHOS2	53.20	1.58	104.83	0.04*
OVTOKO-OVK18	28.54	-23.08	80.16	0.41

*denotes significant difference.

Abbreviations: diff mean, mean difference; lwr, lower end point of the interval; upr, upper end

point of the interval

Supplementary Table 3

Primers used for quantitative RT-PCR.

Gene	Primer	Template-specific sequence (5'-3')	Annealing Temp. [°C]
GGT1	F	GTGTTCTGCCGGGATAGAAA	55
	R	CAGGTCCTCAGCTGTCACAA	
GLS1	F	AGGTGGTGATCAAAGGGTAAAG	54
	R	TCCATGTCCATAGCTGACAAAG	
GPx4	F	GAGGCAAGACCGAAGTAAACTAC	55
	R	CCGAACTGGTTACACGGGAA	
SLC7A11	F	ATGCAGTGGCAGTGACCTTT	54
	R	GGCAACAAAGATCGGAACTG	
GCLC	F	GGAAGTGGATGTGGACACCAGA	53
	R	GCTTGTAGTCAGGATGGTTTG CG	
hACTB	F	CACACTGTGCCCATCTACGA	55
	R	CTCCTTAATGTCACGCACGA	

Abbreviations: F, forward primer; R, reverse primer

Supplementary Figure 1

Time course of fluorescence intensity in CCC, normal ovary and endometrium samples in 12 cases.

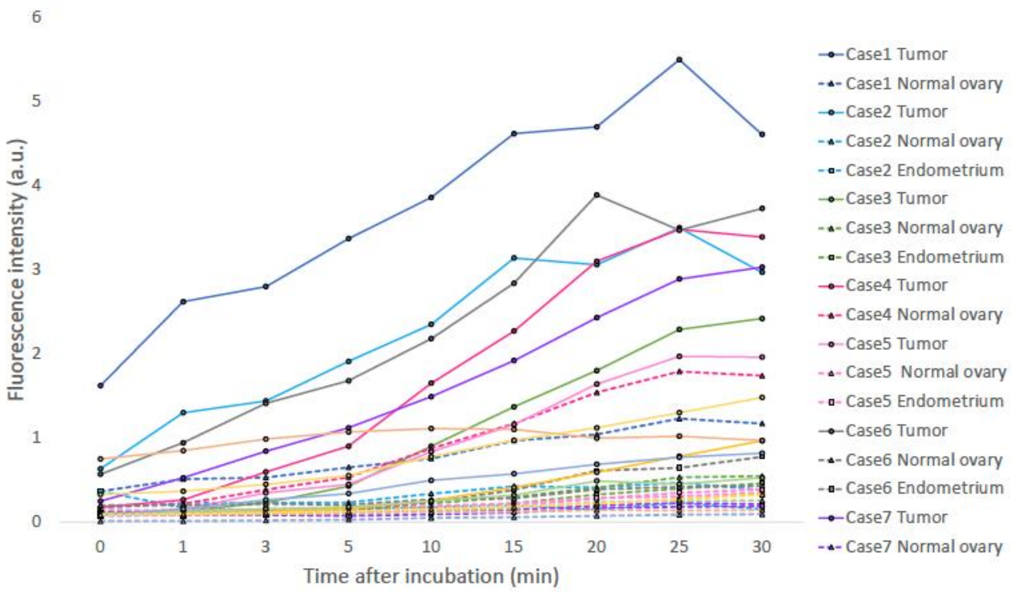

Line colors in the graph indicate each case. Solid lines with circles indicate tumors, dotted lines with triangles indicate normal ovaries, and dotted lines with squares indicate endometrium.
